# Medico-Chirurgical Transactions—Nævi Materni, &c.

**Published:** 1828-04-01

**Authors:** 


### VI.
### MEDICO-CHIRURGICAL TRANSACTIONS.

### 1. *Treatment of Nævi Materni.* Messrs. LAWRENCE and WHITE.

ABOUT 30 pages of the last volume of the Medico-Chirurgical Transactions are occupied with the application of ligature to nævi materni. There is such an expansive principle in medical writings generally, that one would be almost tempted to conclude they were manufactured by steam. Be this as it may, a very useful occupation is that of throwing cold water upon these gazeous productions, and condensing them back within their natural limits. Mr. Lawrence, in particular, keeps a German steamer, impelled by a high-pressure engine, of magnificent dimensions. In this, as in another article on dislocations of the vertebræ, Mr. L. has steamed it among the German rivers of medical literature to some purpose, and brought home a vast cargo of erudition on MUTTERMAHLE —MUTTERFLECKEN—TELANGIEKTASIES—BLUTSCHWAMM, and other jaw-breaking names by which nævi materni have been christened by our learned German neighbours. But, leaving the literature of these nævi on one side, we shall endeavour to get at the marrow of the matter, the treatment, and exhibit it in as short a compass as possible.

Nævi materni, when of considerable magnitude, and situated on or near important parts, require a treatment different from that which has usually been employed. Excision is generally attended with great hæmorrhage, and cannot, indeed, be always effected. The destruction of these unnatural growths has been attempted by caustic, in imitation of the natural sloughing of these parts—and sometimes with success—but this method would be almost as dangerous as excision, where the nævus was large. Our readers are aware that we have given some cases operated on in this way by Mr. Wardrop. Mr. Lawrence has only tried it once, and not with complete success. Cold applications and pressure have been employed by Abernethy, Boyer, and others; but the former is inefficacious, and the latter is generally inadmissible, for obvious reasons. The proceeding adopted by Mr. Wardrop, of tying the arterial trunk or trunks leading to the tumour can seldom be employed with success, though one case terminated favourably in the hands of the proposer. In a case which presented itself in Bartholomew's Hospital, where the size of the tumour rendered excision quite inadmissible, Mr. Lawrence was proceeding to apply caustic, when Mr. Arnott informed him that he had seen Mr. White, of the Westminster Hospital, twice employ the ligature—once at least with success. Indeed the practice was noticed by Mr. Wardrop, in a former volume of the Medico-Chirurgical Transactions. Mr. L. immediately adopted the hint, and the cases operated on in this way form the basis of the present paper. We shall give a condensed view of one or two of these cases.

*Case.* An infant, four months old, presented, at birth, a slight reddish discoloration of the skin, the size of a sixpence, on the back, not elevated. At the end of three months, it began to enlarge—became elevated—changed from red to purple—and frequently discharged blood. It rapidly increased till the age above-mentioned, when it was brought to Bartholomew's. It was now rather larger at the edge than at the base, which rendered the ligature easy. A curved needle was passed under the middle of its basis, armed with a double ligature, and then each thread was passed round its respective semicircle, and tied as tightly as they could be drawn. The infant seemed to suffer a good deal from the drawing of the ligatures, cried almost incessantly for 36 hours, and was occasionally convulsed. Cold applications were used in the mean time. Two days afterwards, Mr. L. sliced off the tumour, which had become almost black—and, on the third day, the ligatures were quite loose. A large slough occupied the centre of the wound, extending deeply, but came away in a few days, under bread poultices. The wound then granulated, and slowly but completely cicatrized.

Three or four other cases are related, all treated in the same manner, and with the same favourable results. It is quite needless to recapitulate them. Mr. L. judiciously advises the ligatures to be drawn as tightly as possible, not only to cut off the supply of blood, but to mortify the offending part with the least delay. Stout silk twist will anwer the purpose. But, as these ligatures necessarily produce considerable irritation, it is desirable to cut them away as soon as they have effected the desired object—the death of the part. This is generally done in 48 hours, if the ligature be properly drawn. The danger of inflammation and irritation is at an end as soon as the ligature is removed.

It is now time to notice the paper of Mr. White, which, though second in order of reading and printing, is evidently entitled to priority, as far as originality and dates are concerned. In another place, we remarked that Mr. Lawrence's reputation will never rest securely on ORIGINALITY—and the present paper is an example. The paper on incisions in erysipelas appeared fifteen years after the practice was proclaimed to the world, by Mr. Hutchison and ourselves. By these observations, we mean not to detract from Mr. Lawrence's merit, but only to shew in what that merit consists. The worst enemy is he who praises a man for qualities which he does not possess—the truest friend is he who gives a fair estimate of an author's talents, nothing extenuating, nor setting down aught in malice. We repeat it, then, that Mr. Lawrence's merit consists in quick apprehension—judicious discernment—patient research—dexterity of hand. Whoever sets him forward as an ORIGINAL GENIUS detracts from his living character, as well as from his posthumous reputation.

Mr. White, in his paper, read on the 12th June, 1827, informs us that it has been his practice, during many years, "to destroy the larger specimens of nævi by strangulation with the ligature:—A curved needle is placed under their base, and a single or double ligature used, according to the magnitude, figure, and situation of the tumour." He had one case, in which it was necessary to pass four ligatures, in order to entangle the whole of the spurious structure, on account of its irregular form and diffused surface, situated behind the ear and extending down the neck of a very young infant. An assistant should raise the tumour, with as much of surrounding skin as possible, thus enabling the operator to pass the needle sufficiently deep, and, at the same time, include within his knot a portion of the healthy integument.

"When the nævus is not large, a needle of sufficient length being carried under the centre of its base, and the structure elevated upon it, a single ligature passed behind the needle, and firmly tied with a double noose, will be sufficient to destroy the organization. Where, however, the size of the tumour renders its strangulation by this mode doubtful, the needle should be armed with a double ligature, to enable the operator to tie one half with each. This, in every instance where the whole substance has been included, has uniformly destroyed the structure, leaving, on its separation, a healthy granulating surface. There are some instances, however, and some situations, where it is impossible to include the whole of the tumour with the ligature; but generally, in such cases, the inflammation excited by its application in the neighbouring parts is sufficient to obliterate, by condensation of the surrounding cellular structure, any remaining portion of the nævus."

Three cases are detailed by Mr. White in illustration, but they can add nothing to the intelligibility of the operation, the utility of which now requires no proofs. Mr. White looks on these nævi as possessing two modes of existence—active and passive—the latter being generally known by the vulgar appellation of strawberries, raspberries, &c. according to the kind of fruit for which the mother longed during utero-gestation. The active form of nævus is a much more formidable affection, and has been denominated "aneurism by anastomosis." "It is a congeries of large blood-vessels, having the structure of veins, rather than of arteries, not having any perceptible arterial action." He has seen some varieties very much resembling the spleen in structure.

From these papers, it appears that Mr. White was the first to put in practice the treatment of nævi materni by ligature—and, consequently, that to him belongs the merit, whatever it may be, of the practice. To Mr. Lawrence the public are much indebted, for corroborating the treatment employed by Mr. White, and also for making that practice public. A valuable remedy is of comparatively little value while confined to the possession of the inventor. In such a case, the publication is really of more importance to society than the discovery.

### 2. *Mr.* BIRCH *on Laceration of the Uterus.*

Two cases of this terrible accident have recently been published by Mr. Birch, lecturer on midwifery at Bartholomew's Hospital—and as one of the patients lived eight weeks, and the other entirely recovered, they are deserving of notice.

*Case* 1. This female, aged 25, fell into labour of her third child, and, after the membranes had given way, and the head had descended into the upper aperture of the pelvis, (the pains still continuing strong) felt a sudden distention of the abdomen, with sense of suffocation. The pulse quickly rose to 140—the uterine pains diminished—but the head did not retreat. Dr. Conquest was summoned, and delivered by the perforator and craniotomy forceps. The delivery was followed by peritoneal inflammation, and she gradually sunk, at the end of eight weeks from the accident.

On dissection, the intestines were found glued together, and much of the peritoneum resembled the pigmentum nigrum. A longitudinal laceration was found in the posterior part of the cervix uteri, and posterior parietes of the vagina, an inch and a half long. Each extremity of the laceration had healed to a slight extent, but the edges of the wound were without granulations.

*Case* 2. This female was 29 years of age, and had borne three children. On the evening of the 16th April, Mr. Birch was summoned to the assistance of Mr. Thorne, who was in attendance from seven o'clock that morning. The membranes had given way—the funis and a foot had presented, and the head and hand were discovered at the upper aperture of the pelvis. Attempts were made to carry up the funis and bring down the foot, but without success. Between two and three o'clock, the woman experienced a violent pain, and quickly afterwards vomited. From this period, she complained of constant pain and soreness of the abdomen, with oppression at the præcordia. The pulse became rapid, and there was some bleeding from the vagina. The labour pains gradually abated, and soon ceased entirely. When Mr. Birch arrived, the countenance exhibited great anxiety—the respiration was hurried—the voice scarcely audible—pulse under 100—oppressive fulness at the pit of the stomach—constant pain in the abdomen, with great tenderness on pressure, especially in the left iliac region. The head was felt at the upper aperture of the pelvis, but by no means wedged within it. Mr. B. arrived at the conviction that the uterus was ruptured, and determined to deliver. The cranium was perforated; but on attempting to expand the blades of the instrument, the head receded over the pubes. The hand was immediately passed per vaginam, and grasping one of the feet, brought it down. The nates and trunk were brought through the pelvis with some difficulty. The head could not be brought through before a perforation was made behind the ear. The placenta was quickly removed by Mr. Thorne, and there was no hæmorrhage. Mr. B. passed his hand into the vagina, and came in contact with several coils of intestine, which he returned through the wound, his hand following them into the cavity of the abdomen. Mr. Thorne made a similar examination, and his report corresponds with that of Mr. Birch. We shall not pursue the details of the after-treatment. Inflammation came on, of course, and the proper means were employed to reduce it, including leeches, fomentations, and calomel and opium, carried to ptyalism. At the end of three weeks she had a relapse, which again required the same measures. But she ultimately recovered.

REMARKS. We cannot for a moment doubt, after the above statements, that either the uterus or vagina was ruptured. Mr. Birch acknowledges that his examination of the wound was not so minute as to enable him positively to say whether it was the one or the other. He thinks that the circumstance of the bladder remaining uninjured, is a certain proof that it was the uterus which was torn. What strikes us as somewhat remarkable, was the examination of the wound by Mr. Thorne, after Mr. Birch had pushed the intestines through it, and even carried his hand into the abdomen. We confess that we should not have liked to exercise this second trial, even to secure a witness of the interesting event.

Mr. Birch has appended some practical conclusions, drawn, not from these cases alone, but from a general consideration of the subject of laceration of the uterus. Some of these conclusions we shall notice in a summary way. The first is, that women die of these lacerations either speedily, or more slowly—secondly, that they sometimes recover, where the rent has been in the cervix uteri or the vagina, the reason, he thinks, being, that the intestines or bladder, coming in contact with the wound, prevent "a further effusion of air or blood into the abdominal cavity," and form a kind of temporary bond of union—thirdly, that the instances of recovery after the escape of the child into the abdomen, if left there, are very few, whilst they are much more numerous, where delivery by turning or gastrotomy has been performed. The corollaries are, 1st, That delivery should always be accomplished—2dly, If the child remain in utero, it should be extracted by forceps or lever, if possible—if not, by the perforator, by turning, or other means—3dly, If the fœtus have partially escaped, it is to be brought back, if practicable, without much violence—if not, it is to be delivered through an opening in the parietes of the abdomen—4thly, If the child have entirely escaped into the abdomen, it is to be brought back through the uterine laceration, if practicable, without great force—5thly, Where this is not practicable, gastrotomy is to be had recourse to—6thly, That whatever is done, should be done quickly—bis dat quo dat cito.

For the construction of these rules, and for the drawing of these conclusions, the author claims no originality. They may, nevertheless, be useful in their present condensed form. We are not aware that any material objection lies against any of the precepts delivered by the learned lecturer on this occasion.

